# Modeling Arterial Pulse Pressure From Heart Rate During Sympathetic Activation by Progressive Central Hypovolemia

**DOI:** 10.3389/fphys.2018.00353

**Published:** 2018-04-09

**Authors:** Björn J. P. van Der Ster, Nicolaas H. Sperna Weiland, Berend E. Westerhof, Wim J. Stok, Johannes J. van Lieshout

**Affiliations:** ^1^Department of Internal Medicine, Academic Medical Center, Amsterdam, Netherlands; ^2^Department of Medical Biology, Academic Medical Center, Amsterdam, Netherlands; ^3^Laboratory for Clinical Cardiovascular Physiology, Academic Medical Center, Amsterdam, Netherlands; ^4^Department of Anesthesiology, Academic Medical Center, Amsterdam, Netherlands; ^5^Department of Pulmonary Diseases, Medical Center, Institute for Cardiovascular Research, VU University Amsterdam, Amsterdam, Netherlands; ^6^School of Life Sciences, The Medical School, MRC/Arthritis Research UK Centre for Musculoskeletal Ageing Research, Queen's Medical Centre, Nottingham, United Kingdom

**Keywords:** heart rate, linear mixed effect models, lower body negative pressure, pulse pressure, sympathetic stimulation

## Abstract

Heart rate (HR) has an impact on the central blood pressure (BP) wave shape and is related to pulse wave velocity and therefore to timing and duration of systole and diastole. This study tested the hypothesis that in healthy subjects both in rest and during sympathetic stimulation the relation between HR and pulse pressure (PP) is described by a linear effect model. Forty-four healthy volunteers were subjected to sympathetic stimulation by continuous lower body negative pressure (LBNP) until the onset of pre-syncopal symptoms. Changes in PP and HR were tracked non-invasively and modeled by linear mixed effect (LME) models. The dataset was split into two groups: the first was used for creating a model and the second for its evaluation. Models were created on the data obtained during LBNP. Model performance was expressed as absolute median error (1st; 3rd quantiles) and bias with limits of agreement (LOA) between modeled and measured PP. From rest to sympathetic stimulation, mean BP was maintained while HR increased (~30%) and PP decreased gradually (~20%). During baseline, PP could be modeled with an absolute error of 6 (4; 10) mm Hg and geometric mean ratio of the bias was 0.97 (LOA: 0.8–1.1). During LBNP, absolute median model error was 5 (4; 8) mmHg with geometric mean ratio 1.02 (LOA: 0.8–1.3). In conclusion, both during rest and during sustained sympathetic outflow induced by progressive central hypovolemia, a LME model of HR provides for an estimate of PP in healthy young adults.

## Introduction

In the resting condition an elevated arterial pulse pressure (PP), the pulsatile component of blood pressure (BP), usually reflects decreased arterial compliance specifically in the elderly and is then associated with multiple adverse cardiovascular outcomes. In contrast, when healthy subjects exercise PP increases by the combined effects of an increase in systolic pressure concomitant to the elevated cardiac output (CO) and a reduction in diastolic pressure related to the vasodilatation in the exercising muscles (Lewis et al., 1983; Kim et al., 2015). PP provides prognostic utility beyond that of mean arterial pressure (MAP) (Selvaraj et al., 2016) and respiratory variations in PP relate to the magnitude of the central blood volume or left ventricular preload (Michard, 2005; Bronzwaer et al., 2015). However tracking PP requires a continuous BP signal either through arterial cannulation or through non-invasive BP measurement by plethysmography (Martina et al., 2012).

**Figure 1:**
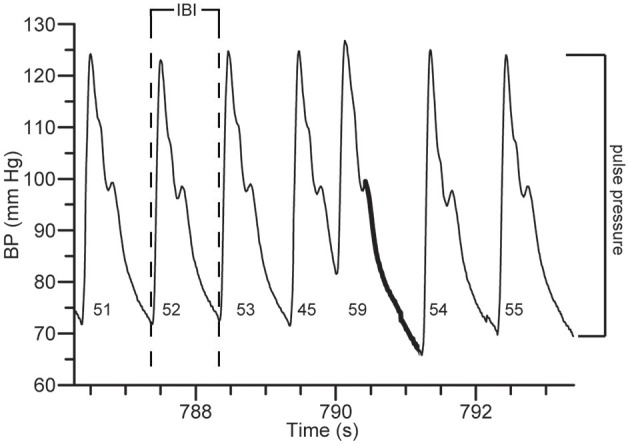
Arterial pressure parameters. Pulse pressure (PP) is indicated for each beat at the root of each beat (in mmHg). Note the effect of increased interbeat interval (IBI) (and thus decreased heart rate) on diastolic pressure runoff (indicated in bold) increasing PP. The preceding beat shows the reverse effect: due to a premature ventricular contraction (reducing the IBI, increasing heart rate), PP decreases.

The duration of the cardiac cycle determines the length of diastole and influences the shape of the arterial pressure waveform (Wilkinson et al., 2000; Lantelme et al., 2002; Albaladejo et al., 2004; Haesler et al., 2004; Westerhof et al., 2008; Mackenzie et al., 2009; Benetos et al., 2010; Westerhof and Westerhof, 2013; Rimoldi et al., 2016). Recently, studies in both animals and humans using pacing or a selective negative chronotropic agent alleged evidence for an inverse relationship between PP and heart rate (HR) (Lantelme et al., 2002; Albaladejo et al., 2004; Haesler et al., 2004; Rimoldi et al., 2016). A lower HR prolongates diastole more than systole, widening PP (Figure 1; Folkow and Ely, 1998).

This study tested the hypothesis that in healthy subjects both in rest and during sympathetic stimulation the relationship between HR and PP can be described by a linear mixed effect (LME) model. To that purpose we tested this in young healthy adults by validating the resulting model on measured PP. Changes in HR and PP were introduced by sympathetic stimulation through induction of progressive central hypovolemia by lower body negative pressure (LBNP).

## Materials and methods

This study was carried out in accordance with the recommendations of Academic Medical Centre Amsterdam medical ethical committee with written informed consent from all subjects. All subjects gave written informed consent in accordance with the Declaration of Helsinki. The protocol was approved by the Academic Medical Centre Amsterdam medical ethical committee (Study no. #2014_310).

### Subjects

Forty-four healthy, non-smoking volunteers (21 females) who performed regular exercise and that did not use cardiovascular medication participated in the study (age: 24, standard deviation (SD) 4 years; height: 177, SD 10 cm; weight: 73, SD 11 kg). Exclusion criteria were a medical history of cardio- and/or cerebrovascular disease, neurological disorders, diabetes mellitus, regular fainting and the use of medication. Prior to the experiment subjects abstained from heavy exercise, alcohol and caffeinated beverages for at least 12 h.

### Instrumentation

Continuous beat-to-beat BP was measured non-invasively using finger plethysmography (Nexfin, Edwards Lifesciences, Irvine, CA, USA; Martina et al., 2012). An appropriately sized finger cuff was applied to the mid-phalanx of the left hand. The hand was maintained at heart level. PP was defined as the difference between systolic and diastolic arterial pressure (SAP and DAP) and HR was defined as the inverse of the interbeat interval. MAP was the mean pressure over one BP pressure wave. Stroke volume (SV) was determined from the arterial pressure pulse wave by Nexfin CO-trek. Nexfin CO-trek readings are not different from a thermodilution estimate of CO for both invasive and non-invasive arterial pressure inputs, nor during upright exercise when compared to inert gas rebreathing (Bogert et al., 2010; Bartels et al., 2011) CO was SV multiplied by HR, and total peripheral resistance the ratio of MAP to CO.

### Protocol

Following instrumentation, the lower part of the body was positioned inside a LBNP box (Dr. Kaiser Medizintechnik, Bad Hersfeld, Germany) and sealed at the level of the iliac crest (Goswami et al., 2009). The LBNP box was equipped with a saddle for subjects to sit on and to prevent leg muscle pump activation during the application of the sub-atmospheric pressure. Following 30 min of supine rest, continuous negative pressure (50 mmHg below atmospheric pressure) was applied to the lower body in one single step. The pressure inside the box was manually controlled and established within 20 s.

### Monitoring of progressive central hypovolemia

Subjects were instructed to minimize movement and maintain their spontaneous breathing frequency and depth. Breathing frequency and end-tidal CO_2_ partial pressure were continuously monitored, verifying that end-tidal CO_2_ did not change significantly during the trial. An investigator experienced in human studies who was unoccupied by other experimental obligations monitored the subjects' well-being. LBNP was terminated either on request, at onset of (pre-) syncopal symptoms or after 30 min. Pre-syncopal symptoms were defined as one or more of the following criteria: systolic arterial pressure below 80 mmHg, or a rapid drop in BP (Δsystolic pressure ≥25 mmHg/min, Δdiastolic arterial pressure ≥15 mmHg/min), a drop in HR of ≥15 bpm, and/or sweating, light-headedness, nausea, vision alterations, skin pallor or a heat sensation.

### Analysis

**Figure 2:**
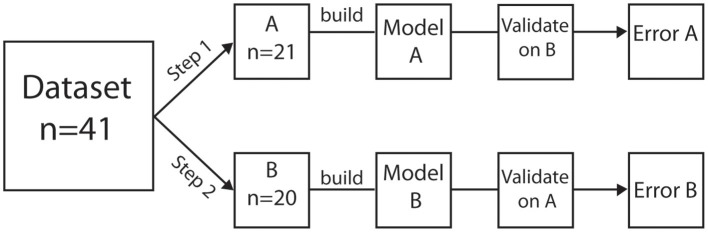
Steps in analysis. Visual representation of the steps required to obtain model performance.

Data collected during the final 10 min of supine rest preceding onset of LBNP was averaged and defined as baseline. Data for each minute during LBNP was averaged and served as model input and output. We constructed LME models describing PP in relation to explanatory variable HR (fixed effect). Subject specific variables (random effects) were included to correct for, subject dependency and the expected variation in HR slope. LME models were computed in R (The R Project, The R Foundation for Statistical Computing, Vienna, Austria) (R Core Team, 2014) using the NLME library (Pinheiro et al., 2014). We used a crossover design to test model performance. The dataset was split into two groups (A and B). Initially, the data of group A served as model input and the data of group B was used to assess the performance of the acquired model. Then the process was interchanged: the data of group B was used to build a model and data of group A to test it (Figure 2). Absolute model errors determined during both baseline and LBNP were assessed per heartbeat. Median errors from all subjects following completion of the interchanging datasets are reported. Except for the modeling all data processing and statistics were performed in Matlab (Matlab 2007b, The MathWorks Inc., Natick, MA, USA).

### Calibration

For the estimation of systolic and diastolic pressure the model was calibrated every 5 min to the 10 beat average of the non-invasive BP monitor to emulate an emergency setting in our hospital first aid department where on a routine basis only intermittent oscillometric BP is available.

### Statistics

A rule of thumb for regression models requires 10 data samples for each included model parameter. Model performance is expressed as absolute median error, bias (mean of differences) and limit of agreement (LOA) (bias ± 1.96 SD) between measured and actual PP. In the case of normally distributed errors, bias and LOA are reported as geometric mean of the ratio between the two parameters. In the case of non-normally distributed model errors a logarithmic transformation was performed on the data before computing bias and LOA followed by a transformation back to get the geometric means ratio and its LOA (Bland and Altman, 1999). Data is reported as means and SD in the case of normally distributed parameters, and as median [1st; 3rd quantile] for parameters that were not distributed normally.

## Results

Data of 3 subjects was excluded because of artifacts or noise in the measured signals, leaving data from 41 subjects available for analysis.

**Figure 3:**
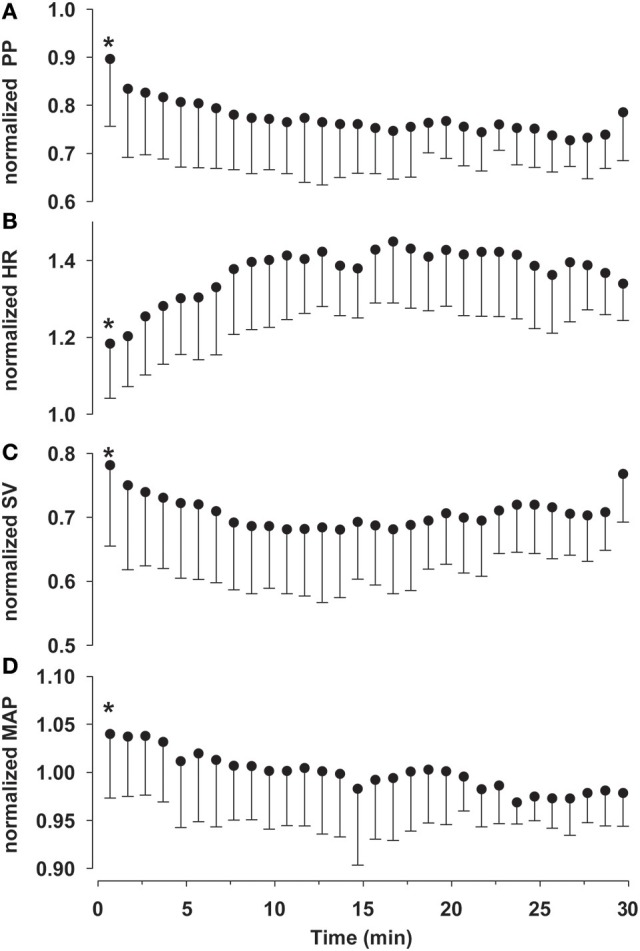
Cardiovascular response to sympathetic stimulation by lower body negative pressure (LBNP). With subjects becoming pre-syncopal the number of subjects within this figure decreases over time. Values normalized to the values in baseline rest preceding LBNP. First minute was tested (paired *t*-test) for statistical significance and noted with an asterisk when different from baseline (*P* < 0.05). **(A)** PP, pulse pressure; **(B)** HR, heart rate; **(C)** SV, stroke volume **(D)** MAP, mean arterial pressure; Mean and standard deviation for each minute during LBNP.

**Figure 4:**
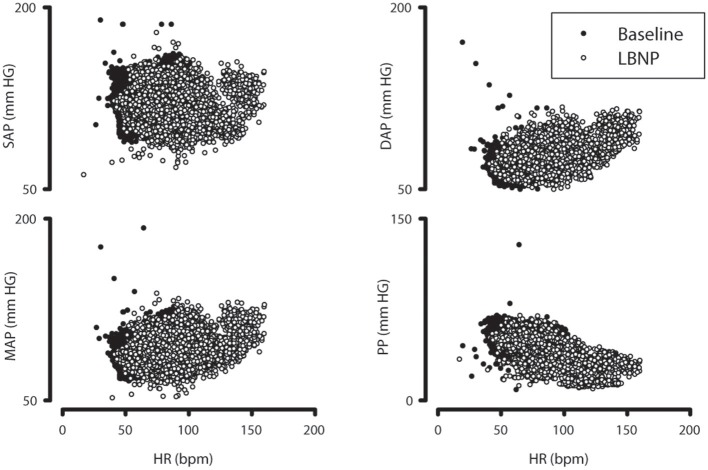
Relationship between heart rate and systolic, diastolic, mean and pulse pressure during LBNP. Scatter plots of the raw data of systolic, diastolic, and mean blood pressure, and pulse pressure vs. heart rate (HR). There is a more distinct decrease of pulse pressure (PP, bottom right) between baseline (filled circles) and during lower body negative pressure (LBNP, open circles) compared to systolic and mean arterial pressure (SAP and MAP).

LBNP induced a rapid decline in SV (~25%) and PP (~15%) accompanied by an immediate ~30% increase in HR (Figure 3). Mean BP was maintained whereas systolic pressure declined ~10% and total peripheral resistance increased ~20%. LBNP was terminated in 37 out of 44 subjects because of pre-syncopal symptoms. Mean time until pre-syncope was 837 (SD 368) s; 7 subjects completed the full 30 min of LBNP. The relationships of HR and the BP parameters during baseline and LBNP (SAP, DAP, MAP and PP) are shown in Figure 4.

### Model validation

The interchanging of datasets resulted in two equations for the model:

(1) $$PP_{A}=67.30-0.35\cdot HR$$

(2) $$PP_{B}=70.04-0.37\cdot HR$$

with HR in beats per minute and PP in mmHg. The distribution of the model input for HR ranged from 58 (SD 9) to 98 (SD 15) bpm. Model within group residuals were 9.0; and 10.3 for group A and B respectively.

**Figure 5:**
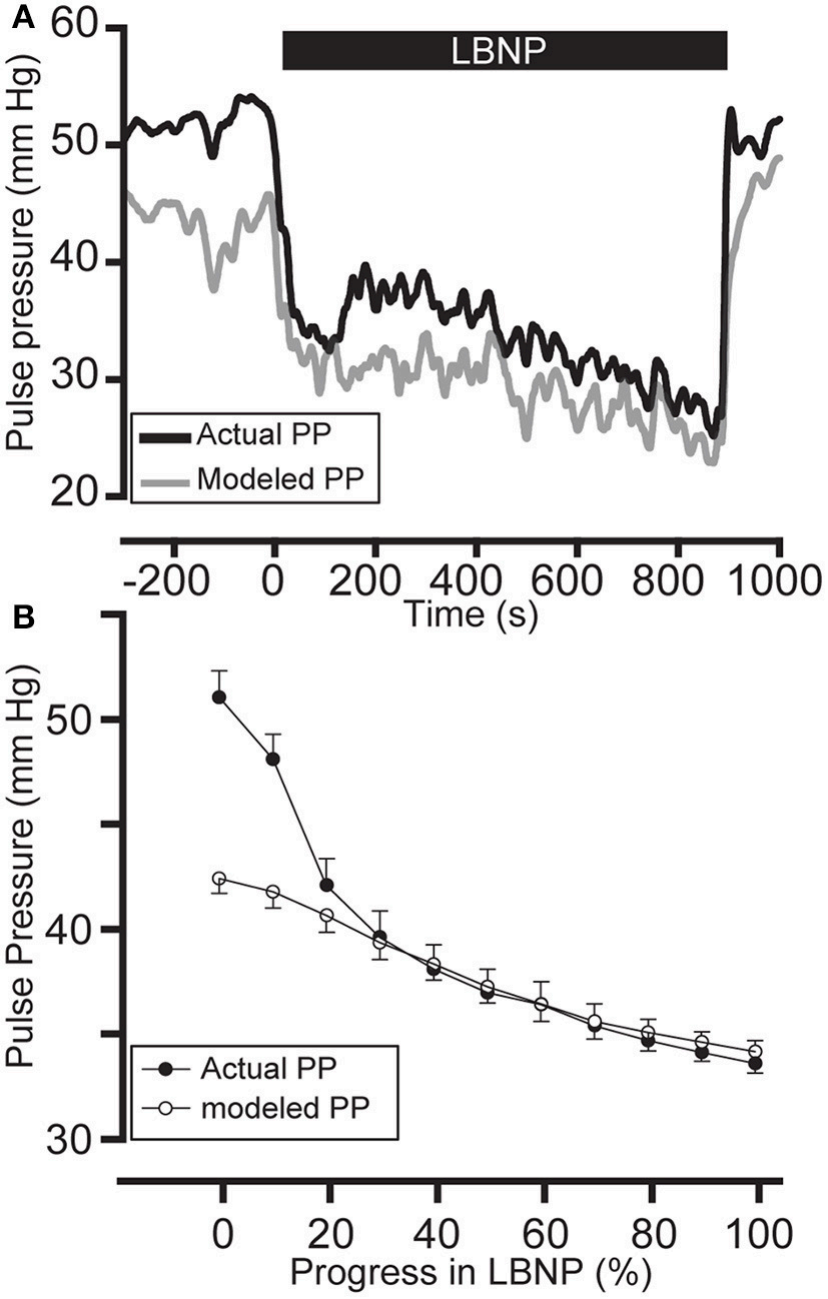
Model performance. Pulse pressure estimated with linear mixed effect model of heart rate in one subject **(A)** and the entire study population **(B)**. Measured (filled circles) and estimated (open circles) pulse pressure (PP) during lower body negative pressure (LBNP). Horizontal axis shows the progress of LBNP in seconds **(A)** and normalized to total duration of LBNP **(B)**, i.e., till pre-syncope or LBNP lasting 30 min. Note that the model misses initial non normally distributed part of the group mean PP due to the onset of LBNP with rapid decrease in PP in the first minute (as noted in Figure 3A). Mean and standard errors in 41 subjects.

Absolute model errors were 6.0 [3.9; 9.8] and 4.9 [3.8; 7.6] mmHg (Figure 5) during baseline and LBNP respectively. Model errors were not normally distributed. Geometric mean ratio of the bias was 0.97 with LOA 0.81–1.13 and 1.02 with LOA 0.77–1.27 during baseline and LBNP respectively.

Calibration of the model to intermittent BP further reduced estimated PP errors to: 2 [2–4] and 3 [2–4] mmHg during rest and LBNP respectively. Errors of systolic and diastolic pressures were normally distributed (Table 1).

**Table 1:** Mean bias, limit of agreement of blood pressure estimation during baseline and lower body negative pressure (LBNP).

	**Baseline**		**LBNP**	
	**Bias**	**Limit of agreement**	**Bias**	**Limit of agreement**
Systole	0.71 [0.3–1.2]	12.39 [11.5–13.2]	1.66 [1.1–2.2]	15.79 [14.8–16.8]
Diastole	0.34 [0.1–0.6]	9.03 [8.4–9.7]	1.47 [1.1–13.9]	14.76 [14.0–15.5]

*Numbers reflect mean and 95% confidence intervals in mmHg*.

**Figure 6:**
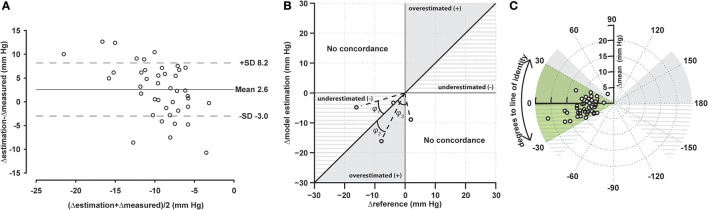
Visualization of the difference in trend assessment during LBNP for both measures of pulse pressure. Bland-Altman **(A)** and polar plot **(B,C)** representation of the difference between the measured and estimated pulse pressure trends. The visualized delta was computed using the mean during LBNP subtracted from the mean during baseline. The angle φ toward the line of identity is either positive (overestimation) or negative (underestimation). φ and the mean of both methods is translated to the polar plot (Critchley et al., 2011) **(C)**.The shaded areas in panels **(B,C)** indicate overestimation of pulse pressure, whereas the hashed areas indicate underestimation of the pulse pressure. The green shaded area indicates the sector including 95% of the samples.

A concordance figure is shown in Figure 6. Mean degree to the line of identity was −5.5 CI 95% [−0.13 to −10.8] degrees. All but one sample fell within the range between −45 and −45 degrees. The sector holding 95% of the samples is ±30 degrees.

## Discussion

The novel finding of this study is that in a setting of a sustained increase in sympathetic outflow by LBNP PP can be estimated from a LME model based on HR. The implication is that in healthy subjects PP can be tracked over time from HR without the requirement of recording continuous (non-)invasive BP. The data indicates that such a model provides an acceptable estimate of PP when venous return becomes reduced with decreasing cardiac volume and filling pressures and enhanced peripheral vascular resistance.

The LBNP procedure was terminated as soon as a subject experienced pre-syncopal symptoms, and the LME models are thus based on a reducing number of samples per passing minute. A merit of LME models is that they allow for such repeated observations and are applicable to datasets with missing data and/or small sample size (Verbeke and Molenberghs, 2000; Gueorguieva and Krystal, 2004; Torng et al., 2007; Pan et al., 2012). Related to the linearity of the equation, our model can only describe the response as described here and not when the relationship between PP and HR reverses, which may occur during pre-syncope where regularly PP and HR decline rapidly. Since PP is inversely modeled to HR (Equation 1), a reduction in HR will incorrectly predict an increase of PP. A restriction of linear models is that they cannot describe such complexity and therefore become unreliable under conditions where the PP—HR relationship is not in agreement with the model's assumptions, i.e., is an inverse linear one.

### Limitations

By design, all subjects were young, healthy individuals, which restrains us from extrapolating the data to aging patients with cardiovascular disease. The model was tested for a limited range of HRs during rest and LBNP and it is unlikely that the model maintains reported performance far beyond the reaches of the used range. The modeled PP (Figure 5B) has difficulty in predicting PP at the start of the LBNP protocol. Due to the almost stepwise fashion in which PP and SV reduce in response to LBNP, the model inherently cannot describe PP accurately at this early stage. Calculating a mean value during such a rapid change is invalid due to the non-normally nature of PP during this phase. This increases estimation error for the initial segment. In a real-life clinical setting, the occurrence of such a rapid drop in blood volume is unlikely.

### Clinical perspective

First line monitoring of patients presenting with hemorrhage includes measuring HR, arterial oxygen saturation and non-invasive intermittent oscillometric blood pressure (NIBP). However, none of these represent biomarkers highly sensitive to the detection of a blood volume deficit since during the initial phase of hypovolemic shock mean BP is maintained by a baroreflex mediated increment in HR and total peripheral resistance (Grant and Reeve, 1941; Barcroft et al., 1944; McMichael, 1944; Ryan et al., 2012; Secher and Van Lieshout, 2016). In contrast, PP decreases progressively during hypovolemic shock and rather than mean BP reflects central blood volume due to its relation to SV (Convertino et al., 2006; Bighamian and Hahn, 2014). Since PP is an important biomarker for changing volume state, application of the reported model could assist in the monitoring of patients at risk for blood loss or development of hypovolemia. It is the rate of change (or trend) noticeable in HR and PP rather than their absolute values that contain the dynamic information essential for clinicians to decide whether the cardiovascular system is in steady-state or is still subject to change. PP is more closely related to flow parameters such as SV where a reduction in PP strongly suggests a decline in SV. In general, BP is not sensitive for a reduction in flow, as illustrated in Figure 3: PP, SV and HR all change during LBNP, whereas MAP is still between 95 and 100% of its initial value.

The presented model does not require additional devices or measurements to function and can be used whenever HR is available, e.g., from electrocardiography or pulse oximetry. Further we demonstrated that the model can upgrade intermittent oscillometric BP to a continuous tracking device of systolic and diastolic BP by a 5 min interval calibration.

In conclusion, in healthy young adults who progress from a resting situation to a situation of sympathetic activation by progressive central hypovolemia PP and its trend can be estimated from HR.

## Author contributions

Conception and experimental design: BS, BW, NS-W, JL, and WS; Data acquisition: BS and WS; Analysis: BS; Figure preparation: BS and JL Manuscript editing: BW, NS-W, WS, and JL.

### Conflict of interest statement

BW previously worked for Edwards Lifesciences Corp. They, however, had no say in any of the data or work presented. The other authors declare that the research was conducted in the absence of any commercial or financial relationships that could be construed as a potential conflict of interest.
